# Open-source software package for on-the-fly deskewing and live viewing of volumetric lightsheet microscopy data

**DOI:** 10.1364/BOE.479977

**Published:** 2023-01-23

**Authors:** Jacob R. Lamb, Edward N. Ward, Clemens F. Kaminski

**Affiliations:** Department of Chemical Engineering and Biotechnology, University of Cambridge, Cambridge, UK

## Abstract

Oblique plane microscopy, OPM, is a form of lightsheet microscopy that permits volumetric imaging of biological samples at high temporal and spatial resolution. However, the imaging geometry of OPM, and related variants of light sheet microscopy, distorts the coordinate frame of the presented image sections with respect to the real space coordinate frame in which the sample is moved. This makes live viewing and practical operation of such microscopes difficult. We present an open-source software package that utilises GPU acceleration and multiprocessing to transform the display of OPM imaging data in real time to produce a live extended depth of field projection. Image stacks can be acquired, processed and plotted at rates of several Hz, making live operation of OPMs, and similar microscopes, more user friendly and intuitive.

## 1. Introduction

Fluorescence microscopy is a key tool for biological research. Continual development of more advanced microscopes has helped to drive progress in fields such as cell and developmental biology and in neuroscience [1,2]. Specifically, the study of rapid cellular and subcellular dynamics requires an ability to take volumetric images, with subcellular spatial and high temporal resolution.

**Fig. 1.**
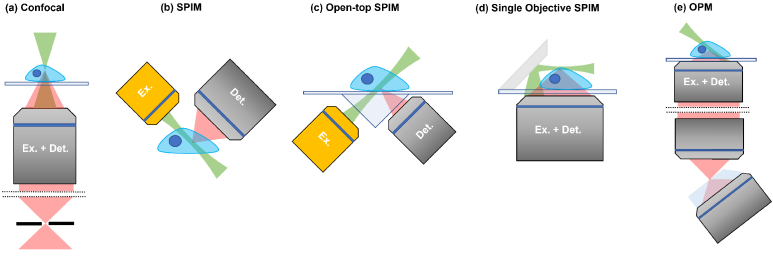
Imaging geometries for optical sectioning microscopy: Optical sectioning is achieved by rejecting fluorescence signal beyond the focal region. In confocal microscopy (a) this is achieved using a pinhole, however, in lightsheet microscopy (b-e) the use of an illumination sheet prevents fluorescence to be generated outside of the focal plane. Conventional two objective based lightsheet modalities (b,c) have restricted spatial resolution due to the requirement for long working distance objectives. Single objective SPIM (d) enables the use of an objective with arbitrary NA, however, the requirement for a mirror in the sample increases mounting complexity. OPM (e) achieves excitation (green) and detection (red) through a single high NA objective placed underneath the sample. Remote refocussing allows the recreation of tilted illumination sheet to be imaged with a tertiary objective at a matching angle.

Selective plane illumination microscopy (SPIM), otherwise known as light sheet microscopy, deploys a novel imaging geometry to allow for 3D imaging with temporal and spatial resolution ideal for probing dynamic processes in full organisms with single cell resolution. In 2004, Huisken et al. detailed the first lightsheet microscope that operated at the diffraction limit [3,4]. In comparison to its most comparable imaging modality, spinning disk confocal microscopy, SPIM not only offers better temporal resolution but also significantly reduces photo-damage and photobleaching in the sample, thus providing more benign imaging conditions over extended durations [5–7]. Consequently, SPIM looked set to replace confocal systems as the imaging modality of choice for volumetric imaging. However, SPIM requires two orthogonal objectives, one to deliver a sheet of excitation light into the sample, and a second to image the resultant fluorescent signal (Fig. 1(b)). As this orthogonal imaging geometry is not usually compatible with inverted sample imaging, where illumination and signal detection take place from underneath the sample, it precludes many of the sample mounting techniques most widely used in biological research, for example the use of multi-well plates and stage mounted incubation chambers [8]. Furthermore, geometrical constraints for positioning the two objectives require that the detection objective has a sufficiently long working distance to prevent its physical obstruction by the body of the illumination objective. This limits the numerical aperture (NA) and thus the achievable spatial resolution is often below that of confocal microscopy. Although SPIM has become the imaging method of choice for large volumetric samples (e.g. whole organisms or 3D tissues), this limitation is the reason the technique has not yet replaced confocal imaging for subcellular, high-resolution studies. In summary, the practical application and adoption of the technology as the method of choice for the majority of biological imaging tasks has remained limited [9].

Variations on the standard SPIM geometry have been developed to address the issues of sample mounting and limitations in usable NA. Open-top SPIM (Fig. 1(c)) uses two orthogonal objectives below the cover glass to allow for the use of standard sample mounting techniques and lab-on-chip type sample containers. However, due to the dual objective geometry, the usable NA remains limited by the requirement for long optical working distances. Furthermore, a long focal length cylindrical lens is required to correct for astigmatism introduced due to imaging at an angle through a glass coverslip [10,11].

Single objective SPIM (Fig. 1(d)) is a variant, where a mirror is mounted across the sample to reflect the excitation sheet along the focal plane of the detection objective. This removes restrictions placed on the usable detection NA. However, the addition of the mirror adds significant complexity to the sample mounting process and requires on-the-fly drift correction to ensure the light sheet remains in focus [12,13].

Oblique plane microscopy (OPM) overcomes these shortcomings and is set to revolutionise SPIM as an ’all-round’ biological imaging technique. In OPM, pioneered by Dunsby in 2008 [14], a single high NA objective is used in epifluorescence mode, and the system is constructed using a standard inverted microscope configuration, a huge advantage for cell biological imaging. The excitation light sheet exits the objective at an oblique angle and the fluorescence signal is collected through the same objective. A second objective lens is then used to recreate a 3D image of the fluorescence using aberration-free remote refocusing [15]. A third objective, at an angle matched to the sheet, is then used to image the slices through the sample (Fig. 1(e)).

Since its original publication, OPM has attracted significant interest, and multiple groups are working on further development of the technique. Much of the effort is directed at improving the spatial resolution of OPM. For example, the use of refractive media between the secondary and tertiary objectives permits the full NA of the primary objective to be exploited by refracting the full cone of fluorescence signal captured by the primary objective into the tertiary objective (achieved via the glass block on the tertiary objective as indicated in Fig. 1(e)) [16,17]. Another improvement concerns the sectioning speed achievable with OPM through use of motorised mirrors [18–20]. However, little progress has been made in improving the operational usability of OPMs via easy real-time data deskewing and visualisation, a key requirement for the method to become widely adopted.

**Fig. 2.**
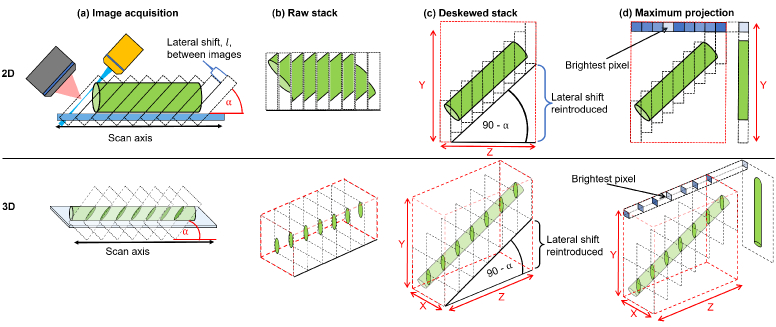
Geometries to achieve optical sectioning and corresponding visualisation of data in lightsheet microscopy: The angle, α , between the excitation sheet and the linear displacement along the scan axis causes a lateral offset between each image section. The sample here is represented by a green cylinder. The coverslip is shown in blue. After acquisition the raw stack is loaded into image visualisation software where the lateral offsets are unaccounted for, and the stack thus represents a distorted geometry of the sample. (b) demonstrates how a cylinder would be represented by a raw stack of lightsheet images. The sample has become distorted, and, in 3D, the cylinder cross sections appear to be stretched, forming an oval shape. To regain the correct shape, the lateral shift must be reintroduced in the deskewing process shown in (c). Then, by taking the brightest pixel across the deskewed stack at each pixel position, a maximum projection can be formed, shown in (d).

When imaging using OPMs and other conventional SPIM systems, the live view for users, when searching for the desired imaging location, is a single angled slice through the sample. The angle between imaging and scanning axes produces image sections that appear distorted with respect to the geometry of the sample (Fig. 2(b)). This view can be very difficult to interpret even for experienced users of lightsheet systems, and often the topology of the imaged sample is not apparent until after a full acquisition has been taken and the results have been deskewed.

Bo-Jui Chang et al. (2021) report on the use of a matched pair of galvanometer mirrors placed in front of the camera that move synchronously with the sample scanning to project a fully deskewed image across the camera chip during a single exposure [21]. Though fast, this method is hardware intensive, and upgrading an existing OPM system is not trivial. To address this issue, we present an open-source Python-based software package that performs rapid data deskewing and projection mathematically, instead of using optics and mechanics. This projection is displayed to the user in real-time, transforming the distorted lightsheet data into an easily interpreted extended depth of field projection.

By using software to perform the deskewing, our method will work on any OPM system, or indeed any lightsheet microscope where the scan direction is at an angle to the focal plane, without the need for additional, or modifications to existing, hardware. The software is designed such that it can perform deskewing in real time whilst image acquisition takes place, in single or multiple colour channels simultaneously. We package our software in an easy to use GUI built using Tkinter, the GUI library included as standard with Python.

The acquisition of a volume stack with a lightsheet microscope requires sample scanning and camera acquisition to occur in synchronicity. The efficiency with which these operations are synchronised directly impacts the achievable temporal resolution. Our software can operate in two modes. In the first, it works in parallel with the existing software controlling the microscope, and this enables for facile and rapid integration into existing platforms. In the second mode, the software handles all hardware synchronisation directly. For the latter case, optimal speed performance can be achieved.

In this article, we begin by illustrating the geometry of lightsheet imaging and describe the image deskewing process required to recover the true geometry of 3D sample structures from raw lightsheet data. We then show how GPU acceleration and multiprocessing permits the transformation of lightsheet data at rates limited only by the microscope hardware. The software significantly improves the usability of OPM and related imaging modalities, thus making the technique more flexible and easier to use for biological researchers, regardless of user expertise.

## 2. Physical principles for processing raw lightsheet imaging data

Each lightsheet image represents an angled slice through the sample with a width, length and depth determined by the illumination and detection optics. The angle of the sheet with respect to the scanning axis induces a lateral offset along the length of the image as the sample is scanned (Fig. 2(a)). This lateral shift, or shear, factor, l , between images is given by

(1) l=zcos⁡(α),

where α is the angle between the sheet and the scan axis, shown in Fig. 2(a), and z is the distance along the scan axis between images. Once a full image stack has been acquired, the data is loaded into image visualisation software, such as Fiji/Imagej [22]. However, this software is unaware of lateral shifts between each image and so the resultant stack distorts the structure of the sample (Fig. 2(b)). In order to recover the correct sample geometry, the lateral shifts between sequential images need to be reintroduced before visualisation. This can be done by an affine transformation [19] or, as is done here, the raw images can be remapped into a larger image of dimensions X and Y (Fig. 2(c)) with the position of each raw image shifting by an amount equal to the original lateral shift between slices. This process is known as deskewing. A maximum intensity projection can then be performed to generate an extended depth of field view of the data. In the software presented here, by remapping each raw image it is possible to build up the final maximum projection as each image is received from the camera. Conversely, the affine transformation method of deskewing would require a full 3D stack to have been acquired before the data processing could begin. Therefore, the remapping method allows for more efficient parallelisation of the tasks of acquiring and processing the raw images.

Due to the detection angle, when the deskewed volume is generated, the sample is reconstructed at an angle to the horizontal equal to ${\left(90-\alpha \right)}^{o}$ , as shown in Fig. 2(c). Consequently, the maximum projection of the stack is a projection of the sample when viewed at this angle. Depending on the morphology of the sample, a more intuitive view may require a perspective from a different angle. Rendering such a view requires a rotation of the image coordinate frame.

**Fig. 3.**
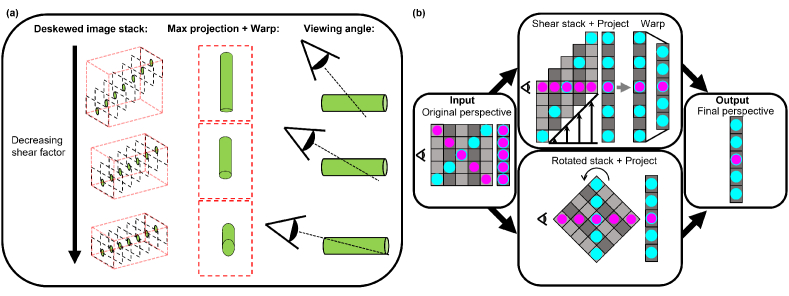
The shear warp algorithm enables rapid rotation of lightsheet volumes: Varying the shear factor ( l , Eq. (1) when deskewing lightsheet data, then warping the final projected image effectively alters the viewing angle of the sample (a). This is because the projection of a sheared and warped volume is equivalent to the projection of the same volume when rotated around its centre (b).

The shear-warp algorithm is used in the field of computer vision as a fast method of rendering the rotation of 3D volumes. It is based on the principle that, for a given perspective, the projection of a 3D volume when rotated about the centre is equal to the projection of the same volume after it has been sheared and then warped, which in the case of the shear-warp algorithm is a scaling along the vertical axis (Y axis in Fig. 2), see Fig. 3(b) [23]. In the context of deskewed lightsheet data, this means that when the shear factor is set according to Eq. (1), the structure of the sample is faithfully reconstructed. However, if the shear factor is reduced and the maximum projection is warped, it has the effect of rotating the projection angle (Fig. 3(a)) [21]. The optical deskewing presented by Bo-Jui Chang et al. (2021) achieve this effect by dynamically adjusting the shear applied by the galvanometer mirror pair. However, here the warping step is neglected. The required warping is equal to cos⁡(θ) , where θ is the angle by which the stack has been rotated. For small angles, the warping step can be ignored, however at larger angles warping is required to faithfully reconstruct the sample geometry. For example, a rotation of 45° requires the image to be vertically scaled by a factor of 0.71.

## 3. Methods for the fast processing and visualisation of lightsheet data

The volumetric imaging rate achievable by OPM is on the order of several Hz, which creates a demand for a method to display deskewed image information to the user at a frame rate sufficient to permit easy and intuitive sample navigation during live imaging.

Fast sample scanning rates can be achieved through use of a high-speed galvanometer scanning mirror. Here, imaging rates are only limited by the acquisition speed of the camera. The problem is now to perform the deskewing process quickly enough to allow for real-time display of the data. Here we show that by utilising multiprocessing and GPU acceleration data can be deskewed and projected at a rate limited only by imaging hardware.

The sequence of operations during imaging involves recording of a stack of images at different scan positions, deskewing each image, calculating the overall maximum intensity projection, and finally displaying the resultant image. During imaging, the live deskewing software needs to control the camera. To ensure widespread support, the package is built on a Python-Micromanager interface (Pycromanager), and thus provides support for the majority of modern scientific cameras [24,25].

To reach display frame rates that are limited only by the imaging hardware, the software must perform the data processing and plotting operations in time intervals shorter than the camera acquisition time. To achieve this goal, we make use of the Python multiprocessing library and split the different computational tasks into separate processes, so that they can run concurrently.

Within the data acquisition process, images are continually taken from the camera and put into a multiprocessing queue ready to be processed. If hardware has been loaded into the software, the scan position is updated after each acquired image (Fig. S3). The images in the queue are stored in RAM however, as long as data acquisition is the slowest process (images are removed from the queue faster than can be put in) the raw data queue will not increase in size over time and the data stored in RAM will remain constant and not exceed that required for a single image. During acquisition, the raw data is not saved to hard disk. Consequently, hard disk speed does not affect the speed of the software.

Data processing presents the most computationally intensive task, thus we employ GPU acceleration to minimise the executing time. Furthermore, the process of deskewing raw lightsheet data involves mapping it into a much larger intermediate data set (Fig. 2(c)). In our software, to minimise the data held in memory, the final maximum intensity projection is updated as each raw image is received. Once received, the raw image is loaded onto the GPU and the maximum intensity projection between it and the region of the final projection it overlaps with, once sheared, is calculated. This process is repeated as each new raw image is received, thus requiring only 3 arrays the size of the raw image (1 for the raw image and 2 needed for maximum projection calculation) and 1 array of the final projected image be stored in VRAM. For example, for a 16-bit raw image of size 256x1024 pixels and projected image of 1024x1024 pixels the total required VRAM is 3.670016MB. Once a full stack has been acquired and processed, the software uses a bilinear interpolation to apply the required warp to the projection and the array is put back into RAM in a multiprocessing queue ready to be plotted (Fig. S4). The user can adjust the desired rotation (changing shear and warp factor) during imaging, giving a ’live’ capability to explore the data from different perspectives (Visualization 2). We achieve a deskewing time of 134.1ms on a typical stack of 200 images to create a 1.96MP projection. This enables users to gain a better understanding of not only the top-down structure of the sample but also of the full 3D structure prior to data acquisition.

The image plotting is performed by a separate thread within the process running the GUI (Fig S5). Multi-colour imaging is performed in a similar way, however, here each raw image is split into the separate colour channels with each channel then distributed to a separate deskewing process. In this way the software does not incur significant loss of performance, even if multiple colour channels are acquired simultaneously, and the software can run at frame rates that are only constrained by hardware limitations.

**Fig. 4.**
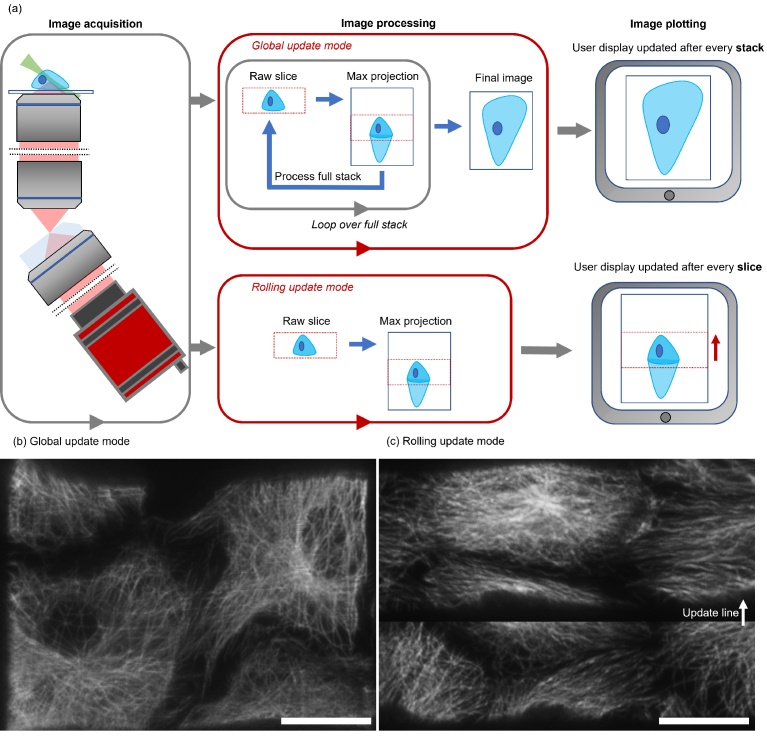
Illustration of two different operational modes of the software to permit operation at either low, or fast imaging acquisition speed. For fast frame rates, global updating of the data stack is used: For slow acquisition speeds a rolling update is used. Camera exposure time is the most influential parameter on software frame rate. (a) Global mode updates the output image after every full stack (Visualization 1 and Visualization 2). However, for long exposure times, this renders the frame rate too low for facile navigation of the sample. Rolling mode updates the output images after each raw image is received from the camera and processed (Visualization 3 and Visualization 4). Global (b) and rolling (c) update modes are shown for the imaging of microtubules in Vero cells. The scale bar is 25um.

Due to the reduced signal, imaging dimmer samples requires the use of longer camera exposure times. The data recorded now comes in so slowly that the live view will refresh too slowly for easy operation of the microscope. We deal with this problem using a so-called rolling update. In this mode, the displayed maximum projection image is updated after every camera exposure on a rolling basis (Fig. 4(a), Visualization 3 and Visualization 4).

Furthermore, when imaging samples where the effect of light scattering is significant, performing the maximum projection of the full volume can lead to poorer contrast reconstructions. To address this we provide functionality in our software to reconstruct a single slice through the sample, rather than projecting the full volume (Visualization 5). To perform this reconstruction, the same data processing pipeline for reconstructing the full volume is used, however, once the raw images are loaded into VRAM all pixel values outside a central horizontal bar are set to zero (Fig S1). The data processing algorithm then only reconstructs signal from the centre of the excitation sheet, with the height of the allowed region determining the depth of the reconstructed slice. For example, a system with a sheet angle of 30° and 115nm pixels, using a central 12 pixel region of each raw image would reconstruct a 1.4 μ m slice of the sample.

## 4. Hardware control and software performance benchmarking

For lightsheet data to be correctly deskewed, the exact lateral shift between each raw image must be known. As can be seen by Eq. (1), this lateral shift is a function of the sheet angle and the lateral distance between each image in the sample. Therefore, prior to data acquisition and deskewing the software must know both of these values. The user must first set the sheet angle and pixel size (effective size of the camera pixel when projected into the sample) in the GUI. The lateral scan distance between each raw image is then calculated as the total field of view (FOV) used divided by the number of images per stack, both of which are user set parameters.

The software can run either in conjunction with a user’s own hardware control software or handle hardware control itself. Should a user wish to use their own hardware control, the step size between each image must be the same as that set in the our software. When sample scanning is controlled in our software, the scan position is controlled via an analogue voltage from a DAQ card. This voltage is either written after each camera frame or, for higher imaging speed, can be preloaded into the DAQ card and triggered externally by the camera (Supplementary note A). Should a user want to use a different method to control the sample scanning we have provided all our the source code and written in a modular form to allow for easy implementation of alternative technologies (Supplementary note B).

The maximum output frame rate of the software is achieved when data processing (deskewing then projecting) and plotting are both faster than data acquisition. In this scenario, the software is limited only by the microscope hardware. The time taken for the three computational processes (acquisition, processing and plotting) are separately dependent on the parameters of exposure time, number of images per stack and FOV along the scan axis (larger FOVs lead to a larger displayed image size), see Table S1.

**Fig. 5.**
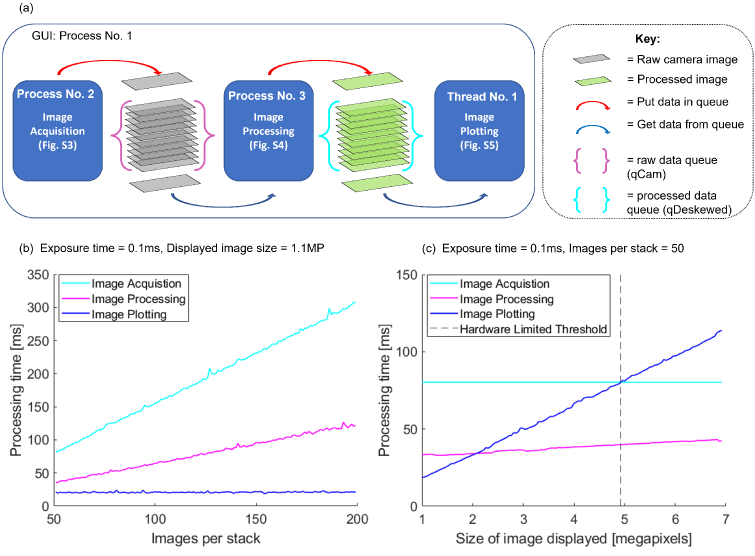
Imaging parameters affect the processing time for the three computational tasks performed by the software during live imaging and reconstruction: Image acquisition, processing and plotting all run concurrently by utilising multiprocessing. Data is transferred between the separate processes via multiprocessing queues, shown in (a). Provided that image acquisition requires the greatest processing time the software runs at a frame rate that is limited by the imaging hardware. The processing time of each computational task is dependant on the on the parameters of exposure time, number of images per stack and final displayed image size. (b) shows the effect of increasing number of images per stack on processing time for a camera exposure time of 0.1ms and displayed image size of 1.1MP. (c) shows the effect of increasing the displayed image size (equivalent to a larger FOV) on processing time for a camera exposure time of 0.1ms and 50 images per stack. In both (c) and (b) a camera region of interest of 1304x87 pixels was used.

For large field of views, the size of the final projected image (see Y in Fig. 2(c)) increases, and both the image processing and plotting tasks require additional processing time. However, if one assumes that the number of images per stack remains constant and instead the step size between images increases, the camera acquisition time remains constant. To test the performance of the software, we experimentally measure the processing time of the different computational tasks during imaging, under different operational parameters (Fig. 5(b,c)). Note that all results presented here are acquired using a Photometrics Kinetix 22 connected via USB 3.2, with the ROI set to 1304×87 pixels, which gives a 150×10 μ m imaging window. Additionally, all results are acquired with scanning controlled via preloaded and externally triggered DAQ card voltages. This mode was chosen as new voltages do not have to be written by the software after each camera frame, thus decreasing the time needed for image acquisition.

Table 1 shows the processing time, in ms, for image acquisition, processing and plotting as a function of displayed image size and number of images per stack, with an exposure time of 0.1ms. An exposure time of 0.1ms was chosen as, with the chosen camera ROI, the exposure time was less than 10% of the readout time. Consequently, image acquisition time could not be meaningfully reduced by decreasing the exposure time. Thus, the presented results for the image acquisition represent the lower bound on the possible acquisition time for the given imaging parameters.

**Table 1.** Processing time, in ms, for Image acquisition, Image Processing and Image plotting as a function of the number of images per stack and the displayed image size, MP, for a camera region of interest of 1304x87 and exposure time of 0.1ms. All presented results are an average of 3 repeats. See Data File 1 for extended results [26].

	No. images per stack		
Displayed image size, MP	40	120	200

0.65	71.3	205.5	377.1
	28.0	74.4	120.8
	11.5	11.5	12.4

1.96	71.3	205.5	377.1
	33.4	79.4	127.8
	35.1	33.2	30.4

3.26	71.3	205.5	377.1
	39.2	84.4	134.1
	55.5	57.4	50.3

For a displayed image size of 0.65MP and 40 images per stack the image acquisition is the slowest process requiring 71.3ms per stack. Increasing the number of images per stack to 200 increases image acquisition and processing time by 429%, to 377.1ms, and 331%, to 120.8ms, respectively. Conversely, image plotting time shows no significant change. This shows that an increase in number of images per stack most strongly affects the acquisition time and the software remains hardware limited as the number of images are increased (Fig. 5(b)). Increasing the displayed image size to 3.26MP increases image processing and plotting time by 11%, to 124.1ms, and 306%, to 50.3ms, respectively. Whereas, image acquisition shows no increase in processing time. As both image processing and plotting time are increasing whilst image acquisition remains constant, further increasing the displayed image size will lead to a condition where the software is no longer hardware limited. For a camera exposure time of 0.1 ms and 50 images per stack, the software remains hardware limited, at 12.5 fps, until the displayed image reaches a size of 4.76MP, at which point image plotting becomes the slower process (Fig. 5(c)). Here, it takes 80ms per stack for both image acquisition (5ms camera exposure, 70ms camera readout and 5ms additional computational time) and plotting.

Decreasing both the number of images per stack and the camera exposure time leads to higher output frame rates. However, altering these parameters can lead to a reduction in the final reconstruction image quality. Using Fourier ring correlation (FRC) as a metric for image quality [27], we measure the software output frame rate and reconstruction image quality as a function of camera exposure time and number of images per stack, for a 0.87MP final displayed image (Table S2). For an exposure time of 25ms and 125 images per stack the software frame rate is 0.3 fps and the FRC resolution is 347.9nm. By reducing the camera exposure time to 1ms, and the number of images per stack to 25, the frame rate can be increased to 12.7fps. However, at the cost of image degradation. With these imaging parameters, the FRC measured resolution becomes 575.8nm. In summary, software frame rate can be increased at the cost of reconstructed image quality.

## 5. Conclusions

We present a data transformation and visualisation tool to enhance the usability of lightsheet microscopes. The software outputs views of projected lightsheet data volumes to facilitate easy sample navigation and general microscope operation. The software is ready-to-use for integration with new, or existing, lightsheet microscopes without need for laborious and costly hardware upgrades. Our method is fully compatible with both high speed galvanometer mirror assisted rapid scanning and slower stage scanned systems. Furthermore, performing volume reconstruction mathematically allows for the use of the maximum intensity projection, which provides a better contrast reconstruction than the summed projection, which is performed when doing real time projection optically.

The software utilises GPU acceleration and multiprocessing to process and display live data at rates limited only by the hardware of the microscope. Choosing between a global and a rolling update mode allows the user to optimise the output frame rate depending on camera exposure time.

We demonstrate the software on a state-of-the-art oblique plane microscope, capable of capturing volumetric image stacks containing 100s of slices at repetition rates of several Hz. However, the software is compatible with all lightsheet modalities where the illumination sheet is at an angle to the scan axis. The source code for the software is freely available on GitHub with full instructions on how to implement and use the system on a specific lightsheet system.

## Data Availability

The source code for the live deskewing software presented here is freely available on GitHub [28]. A static archive of the code used for this paper at the time of publication is also available on Zenodo [29]. Data underlying the results presented in this paper are available in Data File 1, Ref. [26].

## Supplemental document

See Supplement 1 for supporting content.
