# Supplementary material for: Open-source software package for on-the-fly deskewing and live viewing of volumetric lightsheet microscopy data
Source: Biomed Opt Express. 2023 Jan 23;14(2):834–45. doi: 10.1364/BOE.479977 (PMC9979666; doi:10.1364/BOE.479977)
Supplement: Supplementary file 7 [file boe-14-2-834-s001.pdf]

Supplemental Document

Biomedical Optics EXPRESS

Open-source software package for on-the-fly deskewing and live viewing of volumetric lightsheet microscopy data: supplement

JACOB R. LAMB,* EDWARD N. WARD, AND CLEMENS F. KAMINSKI

Department of Chemical Engineering and Biotechnology, University of Cambridge, Cambridge, UK
**jrl70@cam.ac.uk*

This supplement published with Optica Publishing Group on 23 January 2023 by The Authors under the terms of the Creative Commons Attribution 4.0 License in the format provided by the authors and unedited. Further distribution of this work must maintain attribution to the author(s) and the published article's title, journal citation, and DOI.

Supplement DOI: https://doi.org/10.6084/m9.figshare.21896814

Parent Article DOI: https://doi.org/10.1364/BOE.479977

An open-source software package for on-the-fly deskewing and live viewing of volumetric lightsheet microscopy data: supplemental document

1. SUPPLEMENTARY VISUALISATIONS

Videos showing a practical demonstration of our deskewing software on an oblique plane microscope (OPM). Here, the software provides live views of microtubules and actin filaments imaged in Vero cells.

Visualisation 1

In this example the software is operated in global update mode with the extended depth of field reconstruction to image actin filaments. During the video the translation stage is moved laterally.

Visualisation 2

In this example the software is operated in global update mode with the extended depth of field reconstruction to image actin filaments. The translation stage is moved in the z-direction and then the shear warp algorithm is used to give a rotated view of the sample

Visualisation 3

In this example the software is operated in rolling update mode with the extended depth of field reconstruction to image actin filaments. During the video the translation stage is moved laterally.

Visualisation 4

In this example the software is operated in rolling update mode with the extended depth of field reconstruction. The translation stage is moved laterally and the laser is changed to switch from imaging actin to microtubules.

Visualisation 5

In this example the software is operated in global update mode with the single slice reconstruction to image microtubules. The translation stage is moved axially throughout the video showing reconstructions of different slices in the sample.

2. SUPPLEMENTARY FIGURES

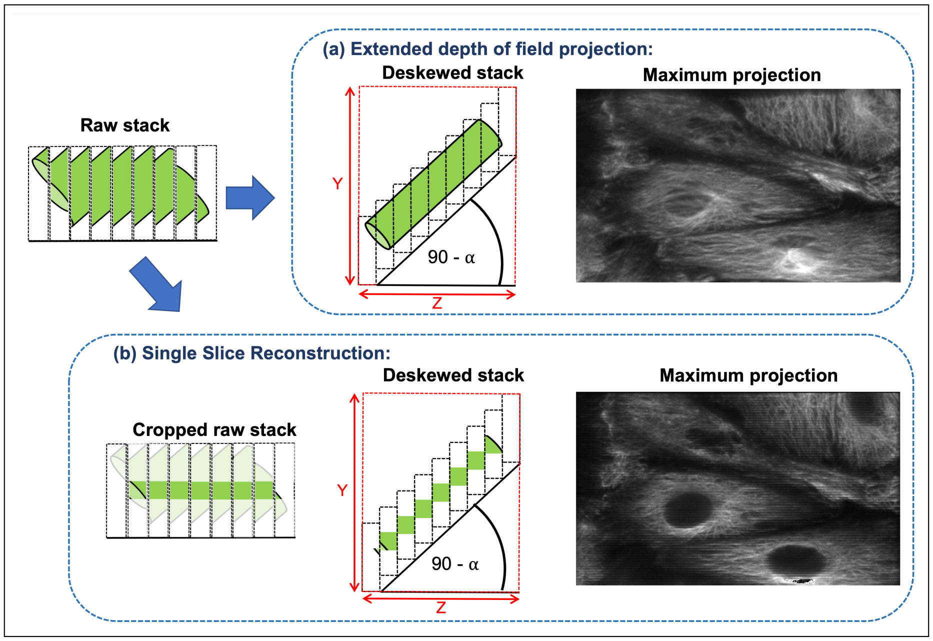

Fig. S1. Cropping raw lightsheet data prior to deskewing and projection reconstructions a single slice of the sample: In samples where light scattering is more prominent maximum projection reconstructions of full volumes suffer from reduced contrast. In this scenario reconstructions of single slices of the sample volume can provide better image quality. This can be achieved by cropping raw lightsheet images to a central horizontal region. Consequently, only signal from the centre of the illumination sheet is retained resulting in a single slice of the volume being reconstructed. The height of the cropped region then determines the depth of the final reconstructed slice with a shorter accepted region leading to a shallower depth of reconstruction. An example of both full volume reconstruction and single slice reconstruction from our software are shown on Vero cell microtubules in (a) and (b) respectively.

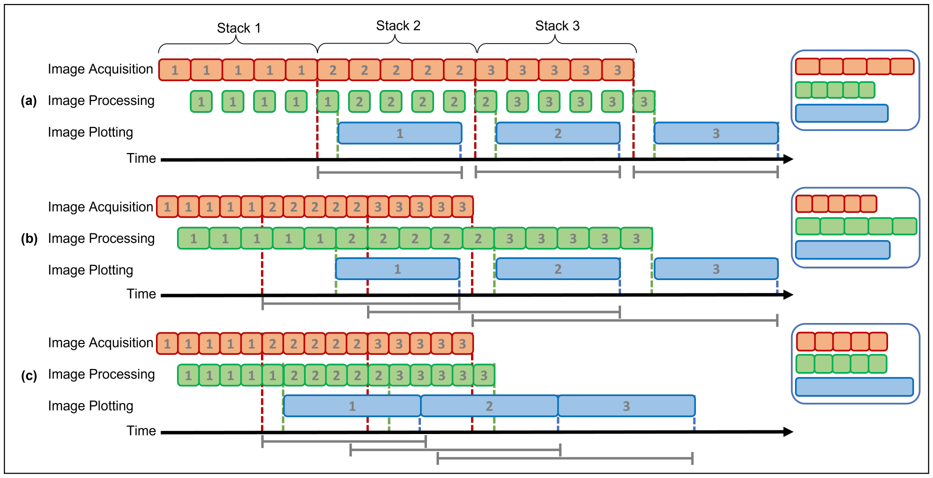

Fig. S2. An illustration of how software performance is affected by image acquisition speed: A stack of raw images must be recorded and processed before the final projection is plotted. The process flow is split into three concurrent processes: image acquisition (orange), image processing (green), and image plotting (blue). (a) When image acquisition is the slowest process, refresh rates for the data display are hardware limited. When either image processing (b) or image plotting (c) are the slowest processes, the camera acquires images faster than the software is able to handle them. Under these conditions, the time between the final image of the stack being acquired and the projection being plotted (grey bar beneath the time axis) grows with each stack. Consequently, the lag time between user navigation and image output increases, resulting in sub optimal user experience.

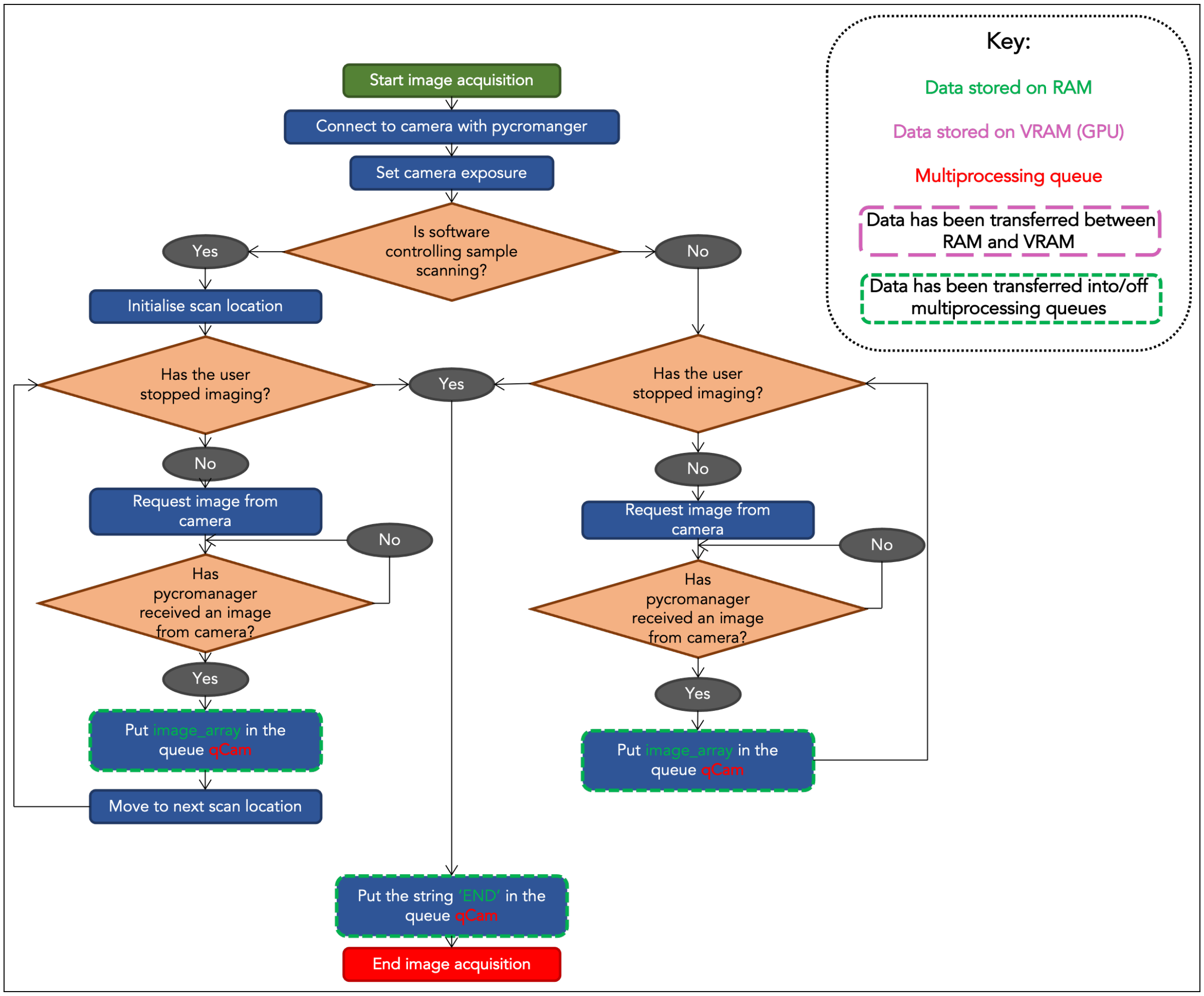

Fig. S3. Flow diagram illustrating the algorithm for data acquisition: Here the algorithm used for data acquisition is illustrated with the data acquired colour coded to show where it is stored, see key. Note that all data stored on multiprocessing queues is also stored on RAM. It is also indicated when data is moved from RAM to VRAM, as well as when data is moved onto and off of multiprocessing queue to illustrate the flow of data within the computer.

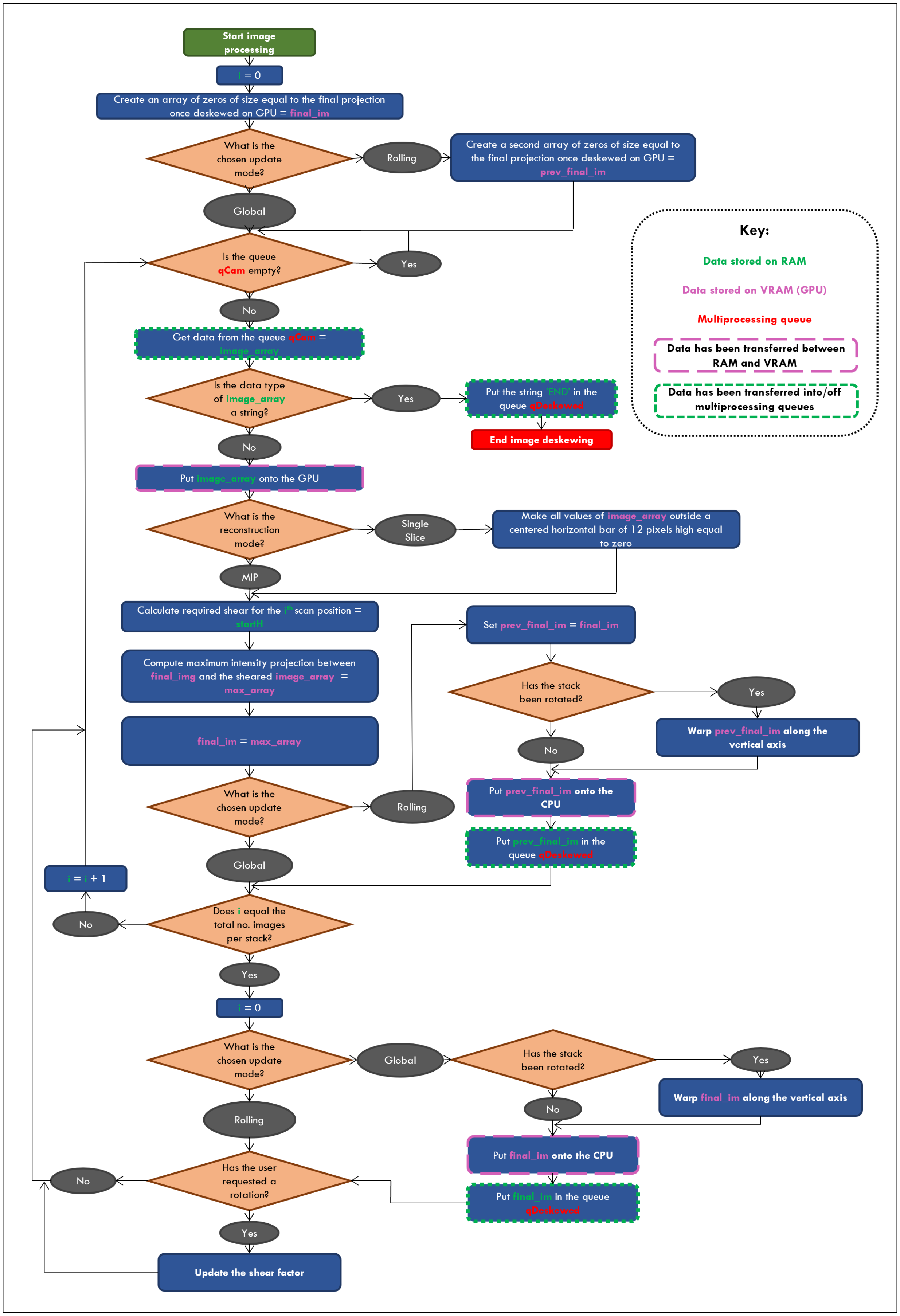

Fig. S4. Flow diagram illustrating the algorithm for data processing: Here the algorithm used for data processing is illustrated with the data acquired colour coded to show where it is stored, see key. Note that all data stored on multiprocessing queues is also stored on RAM. It is also indicated when data is moved from RAM to VRAM, as well as when data is moved onto and off of multiprocessing queue to illustrate the flow of data within the computer.

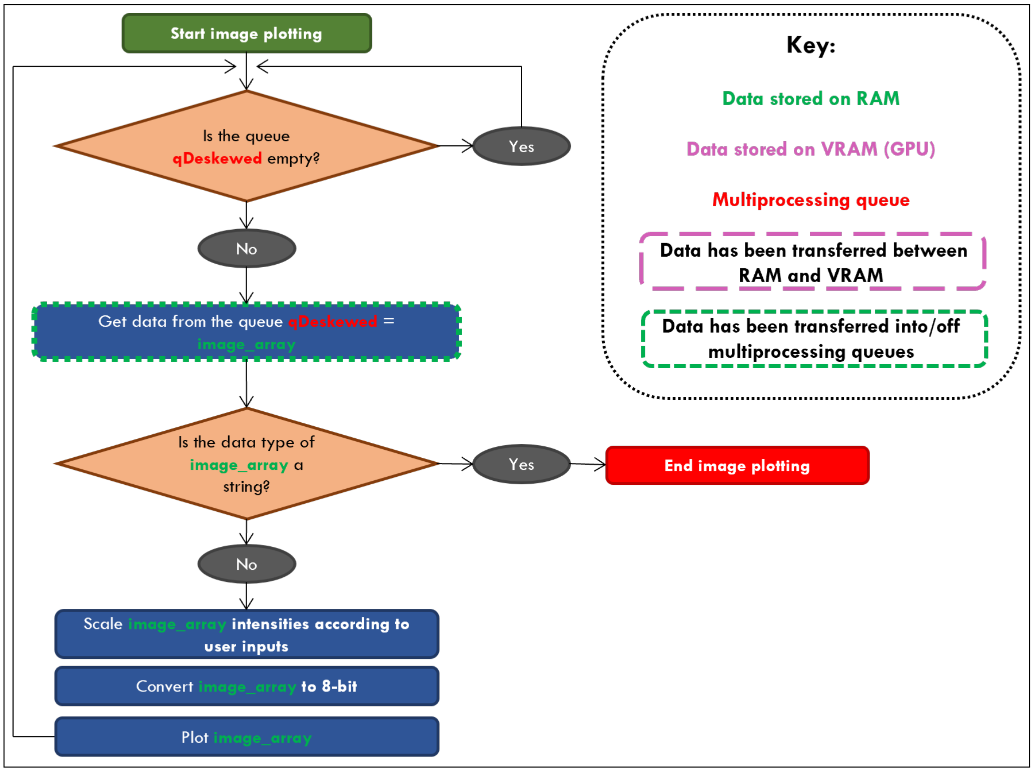

Fig. S5. Flow diagram illustrating the algorithm for image plotting: Here the algorithm used for image plotting is illustrated with the data acquired colour coded to show where it is stored, see key. Note that all data stored on multiprocessing queues is also stored on RAM. It is also indicated when data is moved from RAM to VRAM, as well as when data is moved onto and off of multiprocessing queue to illustrate the flow of data within the computer.

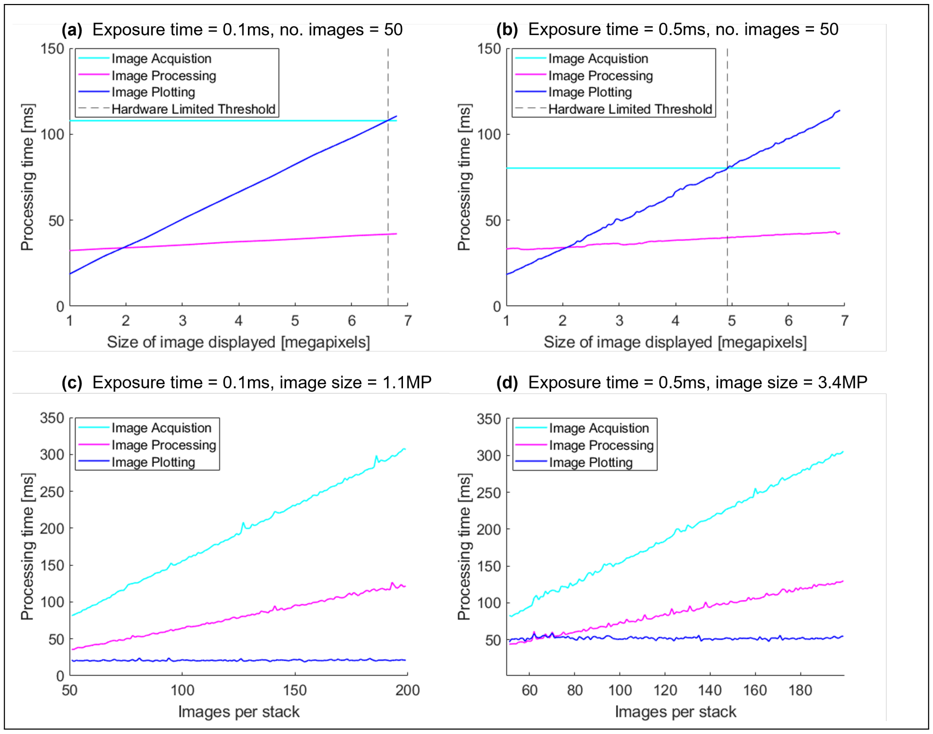

Fig. S6. Software performance only remains hardware limited whilst image acquisition is the slowest process. Here the hardware limited threshold is shown for 4 different sets of imaging parameters: The times required for image acquisition, processing, and plotting each depend differently on the displayed image size, the camera exposure time, and number of images acquired per stack. The software refresh rate remains limited by hardware performance only when the physical image acquisition is the longest task in the process queue. All plots here were produced using a region of interest spanning 1304x87 pixels on the camera. (a,b) For a given exposure time and number of images per stack, image acquisition remains constant, however, image processing and plotting increase linearly. The software stops being hardware limited at an image size of 4.9MP and 6.8MP for exposure times of 0.1ms and 0.5ms, respectively. Increasing the exposure times further would increase the maximum displayed image size where the software remains hardware limited. (c,d) For a given exposure time and image size, image plotting remains constant, however, image acquisition and processing times increase linearly.

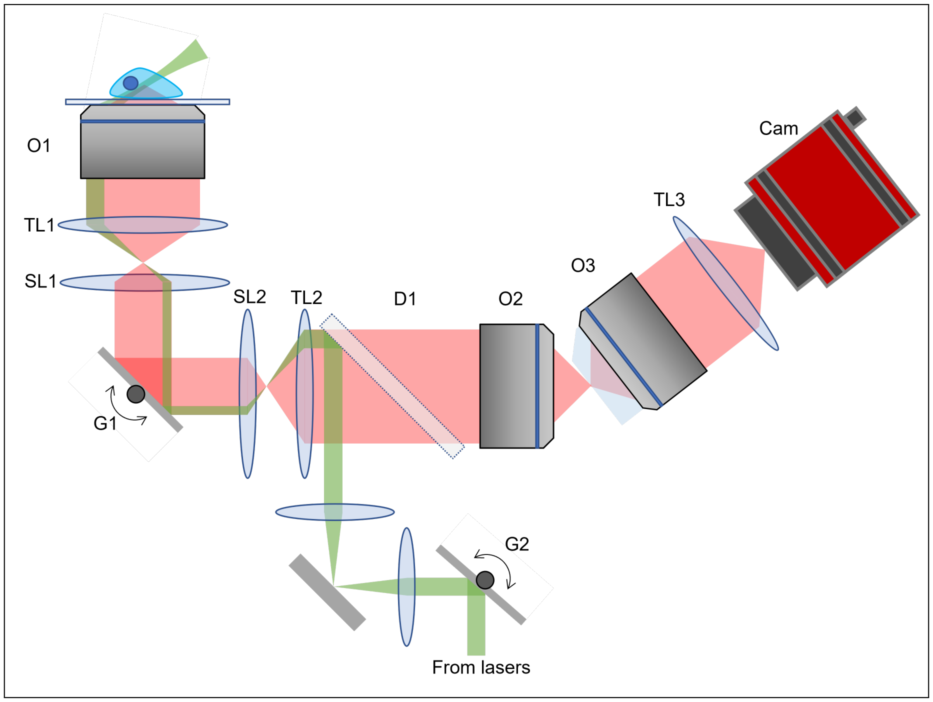

Fig. S7. Optical layout for the oblique plane microscope used in this paper: The primary objective O1 (Olympus UPLSAPO60XS2) collects the fluorescent signal. A tube lens, TL1 (Olympus SWTLU-C), then forms an image of the sample. This image plane is then relayed in a 4f system by two identical scan lenses, SL1 and SL2 (Thorlabs AC-508-075-A and AC-254-100-A combined in a Plossl configuration). In the back focal plane (BFP) at the centre of this 4f system a galvanometric mirror (Scanlab dynAXIS S) is positioned to allow for mirror-based sample scanning. A tube lens with focal length of 193mm, TL2 (combined Thorlabs AC-508-1000-A and TTL200), is then used to perfectly map the BFP of O1 onto the BFP of O2 (UPLXAPO40X objective) to form a remote refocussing system. An angled third objective, O3 (AMS-AGY v1), collects the fluorescent signal and a tube lens, TL3 (Throlabs TTL200), creates an image on the camera (Photometrics Kinetix 22). The sheet is formed by rapidly scanning the focused beam. This is achieved by rotating the beam in a BFP using a galvanometric mirror, G2 (Cambridge Technology 6215H). Note that, though omitted from this diagram for simplicity, a coverslip of thickness of 175μm is used between O2 and O3 to correct for spherical aberrations. The correction collar of O2 is set for a 175μm coverslip and the correction collar of O1 is empirically set on a sample-by-sample basis.

3. SUPPLEMENTARY NOTES

A. Hardware control and synchronisation

The temporal resolution of galvanometer mirror scanning OPM is theoretically limited only by the camera acquisition time. However, without finely optimised hardware synchronisation between camera exposures and galvanometer mirror movements, this theoretical limit cannot be reached, thus losing temporal resolution unnecessarily.

The software has the functionality to control galvanometer mirror scanning, lasers, filters and external camera triggering, in a standalone package to control the full OPM system. Anyone constructing an OPM can make use of it ‘out of the box’ without the need to write complex trigger and synchronisation algorithms.

The galvanometer scan position is controlled by an analogue voltage. This voltage can either be written after each camera exposure or preloaded and externally triggered by the camera. Externally triggering the scan voltages from the exposure line of the camera allows the galvanometer mirror to move and settle during the camera readout time, therefore not limiting the frame rate. Lasers can be controlled by either an analogue or digital signal. Both the filter wheel position and camera triggering are controlled using digital signals. All the hardware can be set up within an

easy-to-use interface and configuration files can be created to allow fast hardware initialisation.

Should the user wish to use their own software to control the hardware of the system, the software can still fully function with any subset of the hardware under its control, provided that the camera is initialised in micromanger. With full access to the source code, users can easily develop support for any other hardware they may require and seamlessly integrate it with the software.

It should be noted that the software does not have any functionality to control multiple cameras. Also, the software does not save any data during image acquisition. This is because, due to the high data acquisition rates of OPMs, in order to save data fast enough as to not affect the software performance the computer would need to be equipped with a specialised high speed storage device.

B. Writing code to control alternative sample scanning hardware

Currently the supported scanning hardware must be controlled via an analogue output from a NI DAQ card. The voltages can either be written after each camera frame or preloaded on the DAQ card and externally triggered.

In order to write support for alternative hardware you will need to amend the code in the file utils/sampleScan.py. Here you can write the functionality to control your hardware. Provided that you ensure that your new code has all the same functions with the same names then there is no need to change any code in any other files.

The class for your hardware must still be called sampleScan. In order to function without any changes to the utils/OPMLiveDeskewing.py code it will also need to have the functions: set_maxV(), set_minV(), set_offV(), setv2um(), set_scanrange(), set_steps(), connect(), createWaveform(), initGalvoPos(), nextPos() and exit().

Here we will describe a suggested sample procedure for amending the sampleScan class to control a motorised stage using serial commands. We provide this example to give the reader an idea of how to go about changing the code, though it should be noted that the steps that we describe are general and could also be applied to other hardwares.

First the user would have to import the Python library Serial. The init function of sampleScan would have to be changed to removed any references to nidaqmax tasks and the input argument of the class should be the com port number where the stage can be found.

The functions set_maxV() and set_minV() are used to set the maximumm and minimum allowed voltages for the galvo to prevent damage. In the case of a stage controlled via serial commands these are not needed. These functions should then remain in the code but be set to not do anything. Alternatively, they can be left as they are however the variables that they reference would be meaningless.

The function set_offV() is used to set the central position of the galvo scan. Similarly to the previous functions this is not required and thus can either be left as it is or made to perform no function.

The function setv2um() is used to set the calibration between voltage applied and sheet movement in microns. Again this would serve no purpose in the case of a motorised stage.

The functions set_scanrange() and set_steps() are used to set the lateral scan distance of the sample and the total number of steps to split this movement up into for each stack. Both of these variables are needed so these functions should be left as they are.

The function connect() is used to open the connection to the DAQ ports. For our example here the serial connection to the stage should be opened at the com port given as the argument to the class.

The function createWaveform() is used to create an array of analogue voltages which the output of the DAQ card loops over. We will need to change this. We will assume that, like the ASI stages, the motorised stage we want to command can be moved by a relative distance in a given axis by sending serial command containing the desired movement distance in microns. In the createWaveform function we will thus change it such that it takes the known scan distance and divides it by the known number of steps per stack. This then give us the desired stage movement after each slice. We will call this variable self.stepsize.

The function initGalvoPos() sets the position of the scan galvo to the first scan position. In the case of a stage we can choose to either have this function do nothing, in this case the area scanned will start at the current stage position, or, it can move the stage by half the amount that will be scanned so that the final scanned area is centred on the current stage position.

The function nextPos() moves the galvo to the next scan location. Here we will change the

functionality so that it sends the appropriate serial command, on the port opened by connect(), to move the stage by an amount equal to self.stepsize. It is also important to note that the deskewing algorithm is written to expect the scanning direction to change after each stack (the volume is scanned back and forth). Consequently, in the function nextPos() we need a counter that updates after each step and when it reaches the number of steps per stack changes the sign of the variable self.stepsize and resets the counter to zero. Depending on the step sizes it might take some time for the stage to reach the desired position. Therefore, it may also be necessary to put a pause in this function to give the stage time to move and settle before the next image is taken on the camera.

Finally, the function exit() will just need to close the serial connection to the stage.

Having made these edits to the file utils/sampleScan.py the software can now perform live deskewing using a motorised stage controlled using serial commands.

C. Cells

Vero cells (from the American Type Culture Collection, CCL-81) were cultured under standard conditions (37°C and 5% CO_2) in Dulbecco's minimum essential medium (DMEM, Sigma-Aldrich) supplemented with 10% heat-inactivated foetal bovine serum (Gibco), antibiotics/antimycotics [penicillin (100 U/ml), streptomycin (100 μg/ml), and amphotericin B (0.025 μg/ml), Gibco], and 2 mM L-glutamine (GlutaMAX, Gibco).

D. Immunostaining

Vero cells were seeded in a glass-bottom 8-well µ-slide (Ibidi, 80827) at a density of 20,000 cells per well and cultured under standard conditions. After 24 hours, cells were fixed by incubation with 4% methanol-free formaldehyde (ThermoFisher Scientific, 28906) and 0.1% glutaraldehyde (Merck, 340855) in cacodylate buffer (100 mM, pH 7.4) for 15 minutes at 37°C. Cells were then washed three times with PBS and permeabilized by incubation with a 0.2% solution of Triton X-100 in PBS for 15 minutes at room temperature. Unspecific binding was blocked by incubating with 10% goat serum (Abcam) and 100 mM glycine in PBS for 30 minutes at room temperature. Without washing, the samples were incubated with an anti-beta-tubulin mouse primary antibody (Abcam, ab131205) diluted 1:200 in PBS containing 2% goat serum for 1 hour at room temperature. After three washes in PBS, the samples were incubated with an Alexa Fluor 568-conjugated anti-mouse IgG1 goat secondary antibody (ThermoFisher Scientific, A-11011) diluted 1:400 in PBS containing 2% goat serum for 1 hour at room temperature in the dark. Samples were then washed 3 times and incubated with a 150 nM solution of ATTO 647N-conjugated phalloidin (Sigma Aldrich, 65906) in PBS for 30 minutes at room temperature in the dark. Cells were then washed three times with PBS and kept in PBS containing 0.05% sodium azide at 4°C until the moment of imaging

E. Computer Hardware

All results presented in the manuscript were obtained running the deskewing software on a computer with an AMD Ryzen 9 5900X 12-Core 3.70 Ghz Processor, 48.0 GB of RAM and a 12288 MiB Nvidia RTX 3060 GPU .

4. SUPPLEMENTARY TABLE

Table S1. Each of the software processes are individually dependent on the user control variables

	Image acquisition time	Image processing time	Image plotting time
Increasing camera exposure time	Increasing	Invariant	Invariant
Increasing number of images per stack	Increasing	Invariant	Invariant
Increasing field of view along the scan axis	Invariant	Increasing	Increasing

Table S2. Image quality variation as function of software input parameters: The output frame rate of the software can be varied by changing the input parameters. The frame rate can be increased by reducing both camera exposure time and the number of steps per stack. However, reducing these parameters has the effect of reducing the reconstructed image quality. Using fourier ring correlation we analyse the reconstructed image quality as a function of no steps per stack and exposure time for a 0.87MP final image. Note that the camera ROI was set to

	No. images per stack			
Exposure time, ms	25	50	75	125
1	12.7<br>575.8	5.8<br>512.9	3.5<br>550.3	2.2<br>546.7
5	6.0<br>424.6	2.7<br>393.6	1.8<br>427.7	1.0<br>377.9
10	3.5<br>398.8	1.6<br>361.7	1.0<br>373.9	0.6<br>355.5
25	1.3<br>370.2	0.7<br>331.9	0.5<br>353.9	0.3<br>347.9

Table S3. Software versions: Here we list all the software that is required to be installed in order to run our software and the versions used for developing the code. Note that any libraries not included here are part of the standard python libraries

Software/Library	Version
Python	3.8.12
Micromanager	2.0.1
LabVIEW	21.0
numpy	1.21.2
nidaqmx	0.5.7
pillow	9.0.1
pycromanager	0.14.1
torch	1.9.1
